# Automated artificial intelligence–enabled measurement of cardiac structures on point-of-care ultrasonography: a prospective multicenter validation study

**DOI:** 10.3389/fcvm.2026.1891962

**Published:** 2026-09-21

**Authors:** Sarah F. Mercaldo, Bernardo C. Bizzo, Tsion Sadore, Madeleine A. Halle, Ashley L. MacDonald, Isabella Newbury-Chaet, Eric L’Italien, Alex S. Schultz, Victor Tam, Sheila M. Hegde, Judy R. Mangion, Praveen Mehrotra, Qiong Zhao, Justina Wu, James M. Hillis

**Affiliations:** 1 Digital Clinical Research Organization, Mass General Brigham AI, Boston, MA, United States; 2 Department of Radiology, Massachusetts General Hospital, Boston, MA, United States; 3 Harvard Medical School, Boston, MA, United States; 4 Division of Cardiovascular Medicine, Department of Medicine, Brigham and Women’s Hospital, Boston, MA, United States; 5 Division of Cardiology, Sidney Kimmel Medical College, Thomas Jefferson University, Philadelphia, PA, United States; 6 Inova Schar Heart and Vascular, Inova Health System, Fairfax, VA, United States; 7 Department of Neurology, Massachusetts General Hospital, Boston, MA, United States

**Keywords:** artificial intelligence, cardiac measurements, echocardiography, machine learning, POCUS, point of care ultrasonography

## Abstract

**Introduction:**

Point-of-care ultrasonography (POCUS) enables clinicians to obtain critical diagnostic information at the bedside, especially in resource-limited settings. This information may include 2D cardiac quantitative data, although measuring the data manually can be time-consuming and subject to user experience. Artificial intelligence (AI) can potentially automate this quantification. This study assessed the interpretation of key cardiac measurements on POCUS images by an AI-enabled device (AISAP Cardio V1.0).

**Methods:**

This retrospective diagnostic accuracy study included 200 POCUS cases from four hospitals (two in Israel and two in the United States). Each case was independently interpreted by three cardiologists and the device for seven measurements [left ventricular (LV) ejection fraction, inferior vena cava (IVC) maximal diameter, left atrial (LA) area, right atrial (RA) area, LV end-diastolic diameter, right ventricular (RV) fractional area change, and aortic root diameter]. The endpoints were the root mean square error (RMSE) of the device compared with the average cardiologist measurement (LV ejection fraction and IVC maximal diameter were primary endpoints; the other measurements were secondary endpoints). Predefined passing criteria were based on the upper bounds of the RMSE 95% confidence intervals (CIs). The intercardiologist RMSE was also calculated for reference.

**Results:**

The device achieved the passing criteria for six of the seven measurements. Although not achieving the passing criterion for RV fractional area change, it still achieved a better RMSE than the intercardiologist RMSE. The RMSE was 6.20% [95% confidence interval (CI): 5.57–6.83; intercardiologist RMSE of 8.23%] for LV ejection fraction, 0.25 cm (95% CI: 0.20–0.29; 0.36 cm) for IVC maximal diameter, 2.39 cm^2^ (95% CI: 1.96–2.82; 4.39 cm^2^) for LA area, 2.11 cm^2^ (95% CI: 1.75–2.47; 3.49 cm^2^) for RA area, 5.06 mm (95% CI: 4.58–5.55; 4.67 mm) for LV end-diastolic diameter, 10.17% (95% CI: 9.01–11.33; 14.12%) for RV fractional area change, and 0.19 cm (95% CI: 0.16–0.21; 0.24 cm) for aortic root diameter.

**Discussion:**

The device accurately calculated these cardiac measurements especially when benchmarked against intercardiologist variability. Its use could assist clinicians who utilize POCUS and better enable their clinical decision-making.

## Introduction

Point-of-care ultrasonography (POCUS) has revolutionized many aspects of acute clinical care in recent decades (1–5). It enables clinicians to obtain more diagnostic information at the bedside, and it has been adopted by multiple specialties, especially emergency medicine and hospital internal medicine (6–9). With some handheld probes connecting directly to a smartphone or tablet, it can be particularly beneficial in resource-limited settings (10–13). Its use includes the quantification of key cardiac measurements such as left ventricular (LV) ejection fraction and inferior vena cava (IVC) size (14).

Artificial intelligence (AI) has similarly started to revolutionize clinical care with many of its current applications relating to medical image interpretation (15, 16). One of its core uses is the automatic quantification of measurements, especially those that otherwise require time-consuming manual tracings. It has previously been used to quantify cardiac measurements using conventional echocardiograms (17–20). It has also been shown to improve interobserver variability and workflow efficiency for echocardiogram interpretation (21, 22). Echocardiography, however, typically provides more comprehensive evaluations with a higher image resolution than POCUS (23).

This study evaluated the ability of an AI-enabled device to calculate seven key cardiac measurements based on POCUS images: LV ejection fraction, IVC maximal diameter, left atrial (LA) area, right atrial (RA) area, LV end-diastolic diameter, right ventricular (RV) fractional area change, and aortic root diameter.

## Methods

### Study design

This diagnostic study was conducted using POCUS cases acquired prospectively from four hospitals in Israel and the United States. The acquisition of cases was approved by local institutional review boards at each of the Baruch Padeh Medical Center, Inova Health, Sheba Medical Center, and Thomas Jefferson University Hospital. It was registered on ClinicalTrials.gov (NCT05631977; first submitted on 17 November 2022). All patients provided informed consent. The subsequent diagnostic study involved transfer of deidentified cases to Mass General Brigham and was approved by the Mass General Brigham Institutional Review Board. The study was conducted in accordance with relevant guidelines and regulations. This manuscript followed the Standards for Reporting of Diagnostic Accuracy (STARD) reporting guideline (24).

### Inclusion criteria

Eligible participants were identified in a consecutive manner in both the inpatient and outpatient settings. They were patients with suspected or established cardiac pathology and with an indication for cardiac ultrasound. They were initially identified by treating physicians such as cardiologists, internal medicine physicians, and emergency department physicians and were consented by local research personnel. Site personnel were trained to acquire cases consecutively. For the first 70% of cases, they recruited all eligible patients. To increase representation of cases with valvular pathology for a related study on valvular pathologies, they were asked to recruit patients only with a known valvular pathology of moderate or severe severity for the final 30% of cases; this request was not specific to any one valvular pathology.

The inclusion criteria included male or female individuals aged 21 or older, the provision of a signed and dated informed consent form, a stated willingness to comply with all study procedures, and availability for the study duration. The exclusion criteria included unstable clinical condition as determined by the principal investigator (where ultrasonography was considered detrimental to the participant), clinical conditions that prevented an ultrasonography study according to the investigators or treating physicians, presence of a prosthetic cardiac valve of any type or position, pregnancy or lactation, contact isolation due to infectious disease, presence of a left ventricular assist device, known congenital heart disease, body mass index (BMI) of 40 or greater, history of lung resection, known very poor cardiac ultrasonography image quality based on prior comprehensive echocardiography examinations, or mechanical ventilation or other situations where the required ultrasonography views could not be adequately obtained.

### POCUS

The POCUS cases were acquired by physicians and sonographers at the bedside using mostly handheld devices (i.e., devices that could connect to a smartphone or tablet). These devices included Kosmos (EchoNous), Lumify (Philips), Navi (Wisonic), and Venue (GE Healthcare); the first two could connect to a smartphone or tablet (25, 26). They contained only four cardiac views according to the American Society of Echocardiography guidelines (27). These views included a parasternal long axis with and without color Doppler, apical four chamber with and without color Doppler, parasternal short axis at the aortic valve level, and subcostal IVC view.

### Cohort selection

Once cases were acquired at individual sites, the cases underwent deidentification prior to transfer to the sponsor. These cases were maintained separately from the cases used for device development. Only two of the four sites (Baruch Padeh Medical Center and Sheba Medical Center; the two sites in Israel) had contributed cases for device development, which had all been acquired prior to the cases used for the validation study. The deidentified cases were subsequently transferred to Mass General Brigham for the diagnostic study. The final cohort required only a subset of cases based on powering calculations, which was selected in a randomized manner.

### Ground truth process

The three ground truth cardiologists (SH, JM, and JW) were US board-certified with a minimum of 5 years of experience in echocardiography. They received training for this project and performed their annotations using a validated custom interface in Siemens syngo.via (version VA20). They reviewed the available loops of the relevant view(s) for each measurement (Supplementary Table 1) and selected the most adequate loop to perform the measurement. They did not receive additional clinical information about the cases. For LV ejection fraction, they visually estimated the measurement; other measurements were calculated based on the American Society of Echocardiography guidelines (27). RV fractional area change was calculated from manually traced end-diastolic and end-systolic RV areas. The component measurements for RV fractional area change were measured three times on different beats by each cardiologist and the average fractional area change was calculated; the other measurements were obtained once per case by each cardiologist. Separately, the cardiologists indicated when a measurement could not be obtained and should therefore be excluded; if at least two of the cardiologists indicated that the measurement could not be obtained, then the measurement was excluded for that case; if only one of them indicated that the measurement could be obtained, then that cardiologist was asked to review the case and make an actual best determination of the measurement.

### Device development and inference

The device evaluated in this study was trained using labeled echocardiography data acquired at the Baruch Padeh Medical Center and Sheba Medical Center between 2009 and 2020. The development set included 145,217 adult patient examinations, which were split into a training set (95,167 cases, 65.5%), validation set (29,090 cases, 20.0%), and a hold-out test set (20,960 cases, 14.4%; which was separate to the cases evaluated in the current study). This development set represented a mix of inpatient and outpatient studies on a range of echocardiography machines. Each examination comprised 20–80 ultrasound images and loops, capturing various cardiac views according to echocardiography protocols. The device incorporated multiple models for different measurements as described below, which included convolutional neural networks (CNNs) and transformers.

For LV ejection fraction estimation, the device used a direct regression model that received apical four chamber and parasternal long-axis views. For both training and inference, the Digital Imaging and Communications in Medicine (DICOM) images for these views underwent preprocessing such as frame extraction, contrast limited adaptive histogram equalization, frame resizing, and tensor conversion. The views were also concatenated. The model sampled 10 frames from each relevant clip over a 1.5-s window. The model architecture combined an EfficientNet-based convolutional backbone, with the classification head removed, for frame-level feature extraction, followed by transformer blocks with multihead self-attention to incorporate temporal information across sampled frames. The resulting features were pooled and passed to a regression head to output a continuous LV ejection fraction value. During training, an L2 regression loss function was used to minimize LV ejection fraction prediction error. To improve robustness across acquisition conditions, augmentations included spatial and image-quality perturbations such as cropping, rotation, sharpness/contrast variation, resolution degradation, and temporal frame jittering. During inference, when more than one valid loop was available for the required view, predictions were averaged across valid loops to improve robustness.

For the remaining measurements, the device used anatomy-specific instance segmentation models followed by rule-based measurement extraction. These models used the appropriate view for the measurement (Supplementary Table 1) and the DICOM images underwent similar preprocessing steps to the LV ejection fraction model. These models were based on a Mask R-CNN with a ResNet-50 Feature Pyramid Network backbone with standard object detection and mask prediction heads. Separate view-specific segmentation models identified relevant anatomical structures such as the aorta, IVC, LA, LV, RA, and RV. The models were optimized using the standard Mask R-CNN multitask loss function, which included region proposal classification loss, region proposal bounding-box regression loss, object classification loss, object bounding-box regression loss, and pixel-wise mask segmentation loss. After segmentation, deterministic postprocessing converted the model masks into the relevant measurements. If there were multiple loops of a view during inference, outliers were removed as applicable and a final measurement was selected using maximum or median functions.

For this study, the device provided a single value for each of the seven measurements. It could also provide an output of “NULL” that reflected it could not interpret that measurement for the case. The reasons included insufficient quality of images (as the device sees them) or inability to calculate the measurement due to the absence of key points in the image used by the device. The device performed inference independently of the ground truth cardiologists and did not receive additional clinical information about the cases.

### Statistical analysis

The statistical analysis was conducted using software R version 4.2.0 (R Foundation for Statistical Computing, Vienna, Austria, 2019). The predefined primary and secondary endpoints were the diagnostic accuracy of the device as determined by the root mean square error (RMSE) when compared with the average of the three ground truth cardiologists (28). LV ejection fraction and IVC maximal diameter were considered primary endpoints, given that these metrics often impact patient care more directly; the other measurements were considered secondary endpoints. The RMSE was calculated as the square root of the mean of the squared differences between the device measurements and the average cardiologist measurements, providing a measure of overall deviation (29). The intercardiologist RMSE was also calculated for reference, which was the average of the pairwise RMSEs between each combination of cardiologists. The passing criteria for the primary endpoints were RMSEs of less than 7.0% for LV ejection fraction and 0.3 cm for IVC maximal diameter, which were to be met by the upper bounds of the 95% confidence intervals (CIs). The passing criteria for the secondary endpoints were RMSEs of less than 20% of the average of the absolute measurement from the three cardiologists, which similarly were to be met by the upper bounds of the 95% CIs.

To further evaluate the interchangeability and accuracy of the device measurements compared with cardiologist measurements, additional predefined secondary assessments were calculated, including the individual equivalence coefficient (IEC; to compare the variability of the relative differences between the device and the cardiologists with the variability of the relative differences between the cardiologists) (30), the mean absolute error (MAE; to assess the average magnitude of errors) (29), the limits of agreement (LOA) using a Bland–Altman analysis (to evaluate the presence of systematic errors between the device and the cardiologists) (31), and the intraclass correlation coefficient (ICC; to measure the consistency and reproducibility of the measurements) (32). The MAE was computed as the mean of the absolute differences between the device and the cardiologist measurements, providing a metric of average error magnitude without being influenced by the direction of the errors. The Bland–Altman analysis involved plotting the mean of each device–cardiologist measurement pair against their difference, allowing for a visual assessment of bias and agreement. The 95% LOA was defined as the mean difference ± 1.96 × standard deviation of the differences, indicating the range within which most differences are expected to lie. For the IEC and ICC calculations, the measurements from each of the cardiologists were considered. For the MAE and LOA, the device was compared with the average measurement from the three cardiologists.

Subgroup analyses were calculated for demographic (sex, age, race, and ethnicity, BMI), disease (LV ejection fraction), and image (ultrasound probe manufacturer, image quality score, role of person acquiring scan) parameters. The subgroups were predefined, except for the race and role of the person acquiring the scan (which were added as *post hoc* analyses), and BMI and LV ejection fraction (for which the subgroups were updated to have more subgroups following regulatory feedback). Each ultrasound probe manufacturer only had a single model, and therefore, both the manufacturers and the models are specified in the results. An additional subgroup analysis was performed as an error analysis following reviewer feedback; it calculated the number and proportion of cases with values within or outside the 95% LOA based on the Bland–Altman analysis for subgroups for LV ejection fraction, ultrasound probe manufacturer, and image quality.

All metrics were accompanied by bootstrapped 95% CIs with 2,000 resamples. An emphasis was placed on the interpretation of CIs to infer the reliability of the calculations. No adjustments for multiple comparisons were made. Sample size calculations were performed *a priori* for the primary endpoints and powered based on preliminary device results.

## Results

### Participant characteristics

The overall cohort included cases from 200 patients (Figure 1). The mean age (± standard deviation) was 65.4 (± 16.0) years and 74 (37.0%) were female patients (Table 1). A case was excluded from the analysis for a given measurement if the cardiologists or the device could not obtain the measurement (Supplementary Table 2).

**Figure 1:**
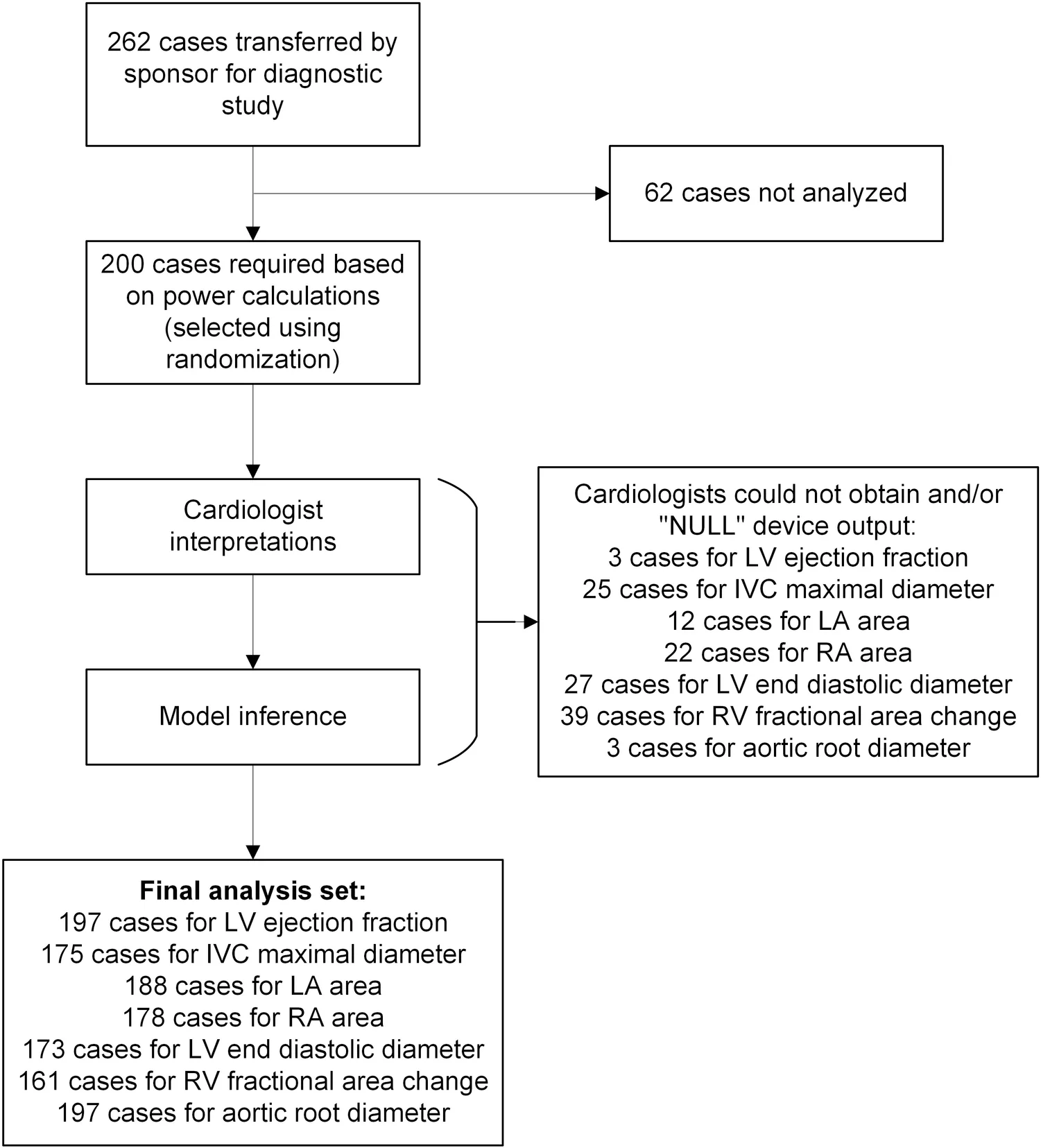
Cohort selection. The 62 cases that were not analyzed reflected that only a subset of cases was required based on powering calculations. IVC, inferior vena cava; LA, left atrial; LV, left ventricular; RA, right atrial; RV, right ventricular.

**Table 1:** Baseline demographic, disease and image information.

Characteristic	Number of cases (%)
Total cohort	200
Age (years)	
≤65	84 (42.0)
>65	116 (58.0)
Mean (± SD)	65.4 (± 16.0)
Sex	
Female	74 (37.0)
Male	126 (63.0)
Race and ethnicity	
American Indian or Alaska Native	2 (1.0)
Asian	11 (5.5)
Black or African American	26 (13.0)
Caucasian	151 (75.5)
Hispanic or Latino	8 (4.0)
Mixed	1 (0.5)
Other	1 (0.5)
BMI	
<25	68 (34.0)
25 to <30	82 (41.0)
30 to <35	37 (18.5)
≥35	11 (5.5)
Unknown	2 (1.0)
Mean (± SD)	27.2 (± 4.7)
LV ejection fraction (%)	
<30	19 (9.6)
≥30 to <53	49 (24.7)
≥53	130 (65.7)
Ultrasound probe manufacturer (model)	
EchoNous (Kosmos)	37 (18.5)
Philips (Lumify)	82 (41.0)
Wisonic (Navi)	25 (12.5)
GE Healthcare (Venue)	56 (28.0)
Image quality score	
1	1 (0.5)
2	2 (1.0)
3	11 (5.5)
4	36 (18.0)
5	99 (49.5)
Unknown	51 (25.5)
Role of person acquiring scan	
Physician	37 (18.5)
Sonographer	183 (81.5)

BMI, body mass index; LV, left ventricular; SD, standard deviation.

### Primary and secondary endpoint analysis

The RMSE was calculated between the device and the three cardiologists (Table 2). Both primary endpoint passing criteria were met, with the RMSEs for LV ejection fraction and IVC maximal diameter having a 95% confidence interval (CI) upper bound of less than 7.0% and 0.3 cm, respectively. Four out of the five secondary endpoint passing criteria were met, with the RMSEs achieving a 95% CI upper bound of less than 20% of the mean cardiologist measurement. RV fractional area change was the one secondary endpoint that did not achieve its passing criterion, although the RMSE was still better between the device and the cardiologists than that between the cardiologists. The other measurements, except for LV end-diastolic diameter, also had better RMSEs between the device and the cardiologists than that between the cardiologists.

**Table 2:** Primary and secondary endpoint results.

Measurement	*N*	Mean measurement (SD)		RMSE (95% CI)	
		Cardiologists	Device	Device versus cardiologists	Intercardiologist
LV ejection fraction	197	53.15% (14.62)	49.92% (12.84)	6.20% (5.57, 6.83)	8.23% (7.52, 8.94)
IVC maximal diameter	175	1.83 cm (0.57)	1.85 cm (0.50)	0.25 cm (0.20, 0.29)	0.36 cm (0.32, 0.40)
LA area	188	21.40 cm^2^ (7.49)	21.63 cm^2^ (6.19)	2.39 cm^2^ (1.96, 2.82)	4.39 cm^2^ (2.88, 5.91)
RA area	178	16.78 cm^2^ (6.74)	16.49 cm^2^ (5.63)	2.11 cm^2^ (1.75, 2.47)	3.49 cm^2^ (3.17, 3.82)
LV end-diastolic diameter	173	45.76 mm (7.71)	49.20 mm (6.98)	5.06 mm (4.58, 5.55)	4.67 mm (4.26, 5.07)
RV fractional area change	161	45.61% (13.12)	41.05% (9.43)	10.17% (9.01, 11.33)	14.12% (13.10, 15.13)
Aortic root diameter	197	3.17 cm (0.47)	3.23 cm (0.40)	0.19 cm (0.16, 0.21)	0.24 cm (0.21, 0.28)

The RMSE between the device and the average of the three cardiologists for the seven measurements (LV ejection fraction and IVC maximal diameter were primary endpoints; the others were secondary endpoints). The intercardiologist RMSE is provided for reference. This table also incorporates the number of cases and mean measurements for each measurement.

CI, confidence interval; IVC, inferior vena cava; LA, left atrial; LV, left ventricular; *N*, number of cases; RA, right atrial; RMSE, root mean square error; RV, right ventricular; SD, standard deviation.

### Secondary assessment analysis

The IEC, MAE, LOA, and ICC were calculated between the device and the three cardiologists (Table 3). The IECs were mostly less than 0, showing better variability of the relative differences between the device and the cardiologists compared with the variability of the relative differences between the cardiologists. The MAEs, which provided an additional statistical method to the RMSEs for measuring the error of the device, were less than the RMSEs. The LOAs, which can show the presence of systematic bias in a device, had mean differences that were unlikely to be clinically meaningful. The ICCs were mostly above 0.75, reflecting the strength of the correlation between the measurements from the device and the measurements from the cardiologists.

**Table 3:** Secondary assessment results.

Measurement	*N*	IEC (95% CI)	MAE (95% CI)	LOA	ICC (95% CI)
LV ejection fraction	197	−0.20 (−0.49, 0.09)	5.01% (4.48, 5.53)	3.18 ± 10.47%	0.84 (0.81, 0.88)
IVC maximal diameter	175	−0.42 (−0.82, −0.03)	0.17 cm (0.14, 0.20)	−0.03 ± 0.48 cm	0.78 (0.72, 0.84)
LA area	188	−0.74 (−1.05, −0.43)	1.67 cm^2^ (1.42, 1.91)	−0.39 ± 4.64 cm^2^	0.84 (0.75, 0.93)
RA area	178	−0.69 (−0.99, −0.40)	1.48 cm^2^ (1.27, 1.70)	−0.03 ± 4.15 cm^2^	0.85 (0.80, 0.90)
LV end-diastolic diameter	173	0.93 (0.35, 1.51)	4.09 mm (3.65, 4.54)	−3.46 ± 7.28 mm	0.77 (0.73, 0.82)
RV fractional area change	161	−0.33 (−0.59, −0.06)	7.91% (6.93, 8.89)	4.77 ± 17.67%	0.42 (0.33, 0.51)
Aortic root diameter	197	−0.16 (−0.61, 0.30)	0.14 cm (0.13, 0.16)	−0.07 ± 0.34 cm	0.86 (0.82, 0.90)

The IEC, MAE, LOA, and ICC between the device and the three cardiologists for the seven measurements. The LOA is expressed as the mean difference ± 1.96 × standard deviation of the differences. This table also incorporates the number of cases for each measurement.

CI, confidence interval; ICC, intraclass coefficient; IEC, individual equivalence coefficient; IVC, inferior vena cava; LA, left atrial; LOA, limits of agreement; LV, left ventricular; MAE, mean absolute error; *N*, number of cases; RA, right atrial; RV, right ventricular.

### Subgroup analyses

The subgroup analyses evaluated the RMSEs across demographic, disease, and image parameter subgroups (Table 4). The vast majority of the RMSEs were similar across subgroups as suggested by overlapping ranges of 95% CIs: there were overlapping ranges for all measurements in the subgroups for the parameters of age, sex, race, and ethnicity, image quality score, and role of person acquiring scan. The measurements with non-overlapping ranges included the RA area for BMI subgroups (the <25 subgroup had a lower RMSE than the [30,35) subgroup), LV ejection fraction for LV ejection fraction subgroups (the <30% subgroup had a lower RMSE than the ≥30% to <53% and ≥53% subgroups), LV end-diastolic diameter for LV ejection fraction subgroups (the <30% subgroup had a lower RMSE than the ≥53% subgroup), and LV ejection fraction for manufacturer subgroups (the GE Healthcare subgroup had a lower RMSE than the Philips or Wisonic subgroups). In addition, the BMI unknown subgroup had only 2 cases and often had non-overlapping 95% CIs.

**Table 4:** Subgroup analysis results.

Subgroup	RMSE (95% CI; *N*)						
	LV ejection fraction	IVC maximal diameter	LA area	RA area	LV end-diastolic diameter	RV fractional area change	Aortic root diameter
Age							
≤65 years	5.61% (4.50, 6.73; 82)	0.26 cm (0.17, 0.34; 74)	2.35 cm^2^ (1.42, 3.29; 79)	2.13 cm^2^ (1.50, 2.76; 78)	4.76 mm (4.04, 5.48; 77)	11.03% (9.22, 12.85; 67)	0.17 cm (0.12, 0.21; 84)
>65 years	6.59% (5.86, 7.32; 115)	0.24 cm (0.20, 0.28; 101)	2.42 cm^2^ (2.05, 2.79; 109)	2.10 cm^2^ (1.68, 2.52; 100)	5.29 mm (4.64, 5.95; 96)	9.51% (8.03, 11.00; 94)	0.20 cm (0.17, 0.23; 113)
Sex							
Female	6.45% (5.44, 7.46; 73)	0.23 cm (0.17, 0.28; 66)	2.46 cm^2^ (1.61, 3.31; 69)	1.78 cm^2^ (1.01, 2.54; 66)	4.60 mm (3.79, 5.40; 65)	9.29% (7.71, 10.86; 59)	0.19 cm (0.16, 0.22; 72)
Male	6.05% (5.26, 6.84; 124)	0.26 cm (0.20, 0.32; 109)	2.35 cm^2^ (1.91, 2.79; 119)	2.29 cm^2^ (1.88, 2.69; 112)	5.33 mm (4.73, 5.92; 108)	10.65% (9.06, 12.25; 102)	0.19 cm (0.15, 0.22; 125)
Race and ethnicity							
Black or African American	7.77% (5.24,10.30; 25)	0.20 cm (0.08, 0.32; 22)	2.05 cm^2^ (1.49, 2.62; 23)	2.19 cm^2^ (1.17, 3.21; 22)	4.37 mm (2.95, 5.78; 25)	9.71% (7.43, 11.98; 19)	0.20 cm (0.15, 0.25; 26)
Caucasian	6.14% (5.54, 6.74; 150)	0.25 cm (0.20, 0.31; 132)	2.36 cm^2^ (1.86, 2.87; 145)	2.07 cm^2^ (1.65, 2.50; 135)	5.34 mm (4.80, 5.87; 126)	9.64% (8.35, 10.93; 124)	0.19 cm (0.16, 0.22; 148)
Other races (combined)	4.38% (3.11, 5.65; 22)	0.24 cm (0.15, 0.33; 21)	2.90 cm^2^ (1.50, 4.30; 20)	2.28 cm^2^ (1.13, 3.44; 21)	4.10 mm (2.53, 5.67; 22)	13.66% (9.33, 17.98; 18)	0.17 cm (0.12, 0.23; 23)
BMI							
<25	6.49% (5.42, 7.57; 67)	0.21 cm (0.16, 0.27; 60)	2.32 cm^2^ (1.78, 2.86; 64)	1.43 cm^2^ (1.09, 1.77; 58)	4.56 mm (3.84, 5.28; 58)	9.15% (7.02, 11.28; 57)	0.19 cm (0.15, 0.22; 67)
(25,30)	6.11% (5.05, 7.17; 81)	0.28 cm (0.20, 0.36; 75)	2.62 cm^2^ (1.79, 3.44; 78)	1.92 cm^2^ (1.47, 2.37; 74)	5.33 mm (4.58, 6.07; 73)	11.39% (9.58, 13.20; 68)	0.15 cm (0.12, 0.18; 81)
(30,35)	6.29% (5.00, 7.57; 36)	0.24 cm (0.17, 0.30; 30)	2.12 cm^2^ (1.61, 2.63; 33)	3.03 cm^2^ (2.05, 4.02; 34)	5.22 mm (3.92, 6.52; 29)	9.37% (6.96, 11.78; 27)	0.24 cm (0.17, 0.31; 36)
≥35	5.01% (3.65, 6.37; 11)	0.16 cm (0.05, 0.27; 8)	2.10 cm^2^ (1.17, 3.04; 11)	2.92 cm^2^ (1.21, 4.64; 10)	5.59 mm (3.44, 7.74; 11)	8.60% (3.03, 14.18; 7)	0.23 cm (0.14, 0.32; 11)
Unknown	4.16% (4.13, 4.20; 2)	0.24 cm (0.00, 0.47; 2)	0.43 cm^2^ (0.08, 0.79; 2)	1.18 cm^2^ (0.55, 1.82; 2)	3.51 mm (2.13, 4.89; 2)	9.53% (2.25, 16.81; 2)	0.03 cm (0.03, 0.03; 2)
LV ejection fraction (%)*							
<30	4.29% (3.24, 5.33; 19)	0.27 cm (0.17, 0.38; 16)	3.45 cm^2^ (1.83, 5.06; 18)	1.78 cm^2^ (1.25, 2.31; 17)	3.57 mm (2.28, 4.85; 14)	10.74% (7.42, 14.06; 14)	0.14 cm (0.08, 0.19; 19)
≥30 to <53	7.04% (5.79, 8.29; 49)	0.18 cm (0.14, 0.22; 41)	1.63 cm^2^ (1.26, 2.00; 46)	1.79 cm^2^ (1.11, 2.47; 45)	4.44 mm (3.53, 5.34; 46)	9.75% (7.35, 12.15; 38)	0.19 cm (0.14, 0.24; 49)
≥53	6.10% (5.33, 6.87; 129)	0.26 cm (0.20, 0.32; 117)	2.45 cm^2^ (1.92, 2.98; 123)	2.27 cm^2^ (1.79, 2.75; 115)	5.46 mm (4.85, 6.07; 111)	10.24% (8.78, 11.71; 109)	0.19 cm (0.16, 0.22; 127)
Ultrasound Probe Manufacturer (Model)							
EchoNous (Kosmos)	5.20% (4.20, 6.21; 37)	0.30 cm (0.15, 0.45; 34)	2.54 cm^2^ (0.99, 4.08; 36)	1.76 cm^2^ (1.40, 2.13; 34)	5.18 mm (4.02, 6.35; 32)	8.50% (6.29, 10.71; 25)	0.15 cm (0.12, 0.19; 37)
Philips (Lumify)	7.12% (6.01, 8.23; 80)	0.23 cm (0.17, 0.28; 70)	1.96 cm^2^ (1.50, 2.43; 74)	1.86 cm^2^ (1.42, 2.31; 71)	5.10 mm (4.32, 5.88; 70)	9.71% (8.27, 11.15; 66)	0.21 cm (0.16, 0.25; 80)
Wisonic (Navi)	7.28% (5.93, 8.63; 25)	0.19 cm (0.13, 0.25; 23)	2.62 cm^2^ (2.00, 3.24; 25)	1.90 cm^2^ (1.22, 2.59; 24)	5.09 mm (3.54, 6.64; 18)	8.77% (5.72, 11.82; 22)	0.18 cm (0.13, 0.23; 25)
GE Healthcare (Venue)	4.67% (3.75, 5.59; 55)	0.25 cm (0.19, 0.31; 48)	2.70 cm^2^ (1.97, 3.43; 53)	2.69 cm^2^ (1.78, 3.60; 49)	4.94 mm (4.11, 5.76; 53)	12.03% (9.51, 14.54; 48)	0.18 cm (0.14, 0.22; 55)
Image quality score							
≤4	5.68% (4.49, 6.87; 48)	0.26 cm (0.18, 0.33; 41)	2.32 cm^2^ (1.79, 2.85; 45)	2.27 cm^2^ (1.76, 2.78; 41)	4.51 mm (3.83, 5.18; 44)	9.07% (6.56, 11.58; 39)	0.19 cm (0.15, 0.23; 48)
>4	5.70% (5.00, 6.40; 99)	0.27 cm (0.19, 0.34; 89)	2.62 cm^2^ (1.90, 3.35; 94)	2.15 cm^2^ (1.53, 2.76; 92)	5.02 mm (4.31, 5.73; 86)	10.88% (9.14, 12.63; 82)	0.18 cm (0.14, 0.22; 98)
Unknown	7.51% (6.02, 8.99; 50)	0.19 cm (0.14, 0.23; 45)	1.95 cm^2^ (1.51, 2.39; 49)	1.88 cm^2^ (1.41, 2.35; 45)	5.66 mm (4.59, 6.73; 43)	9.68% (7.90, 11.46; 40)	0.20 cm (0.16, 0.23; 51)
Role of person acquiring scan							
Physician	5.90% (4.77, 7.03; 37)	0.19 cm (0.14, 0.23; 35)	1.83 cm^2^ (1.38, 2.28; 36)	1.79 cm^2^ (1.33, 2.24; 36)	5.12 mm (3.91, 6.33; 29)	9.48% (6.93, 12.03; 29)	0.20 cm (0.12, 0.28; 36)
Sonographer	6.27% (5.58, 6.97; 160)	0.26 cm (0.20, 0.31; 140)	2.51 cm^2^ (2.02, 3.00; 152)	2.19 cm^2^ (1.74, 2.63; 142)	5.05 mm (4.51, 5.60; 144)	10.32% (9.03, 11.61; 132)	0.18 cm (0.16, 0.21; 161)

The RMSE between the device and the average of the three cardiologists for the seven measurements for demographic, disease, and image parameter subgroups.

* Cardiologist measurements for LV ejection fraction were not available for 2 cases (see Supplementary Table 2), resulting in possible differences in the total N for those rows.

BMI, body mass index; CI, confidence interval; IVC, inferior vena cava; LA, left atrial; LV, left ventricular; *N*, number of cases; RA, right atrial; RMSE, root mean square error; RV, right ventricular.

A subgroup analysis was also performed to determine whether there were subgroups consistently associated with larger errors (Supplementary Table 3). This analysis determined the number and percentage of cases within or outside the 95% LOA based on a Bland–Altman analysis for subgroups for LV ejection fraction, ultrasound probe manufacturer, and image quality. There were no subgroups that had consistent errors (i.e., having a higher or lower percentage of cases outside the 95% LOA compared with within the 95% LOA across all measurements).

## Discussion

This study assessed the performance of an AI-enabled device at interpreting seven key cardiac measurements on images acquired using POCUS devices (Figure 2). It achieved both of its primary endpoint passing criteria and all but one of its secondary endpoint passing criteria, which were based on the RMSE between the device and the average of three cardiologists for the measurements. It also demonstrated better performance compared with the intercardiologist RMSE for all but one of the measurements. Importantly, this study was based mostly on images acquired from inexpensive, predominantly handheld devices that could perform a short POCUS scan at the bedside unlike many other studies that have used images from comprehensive echocardiography examinations obtained in echocardiography laboratories (17–19).

**Figure 2:**
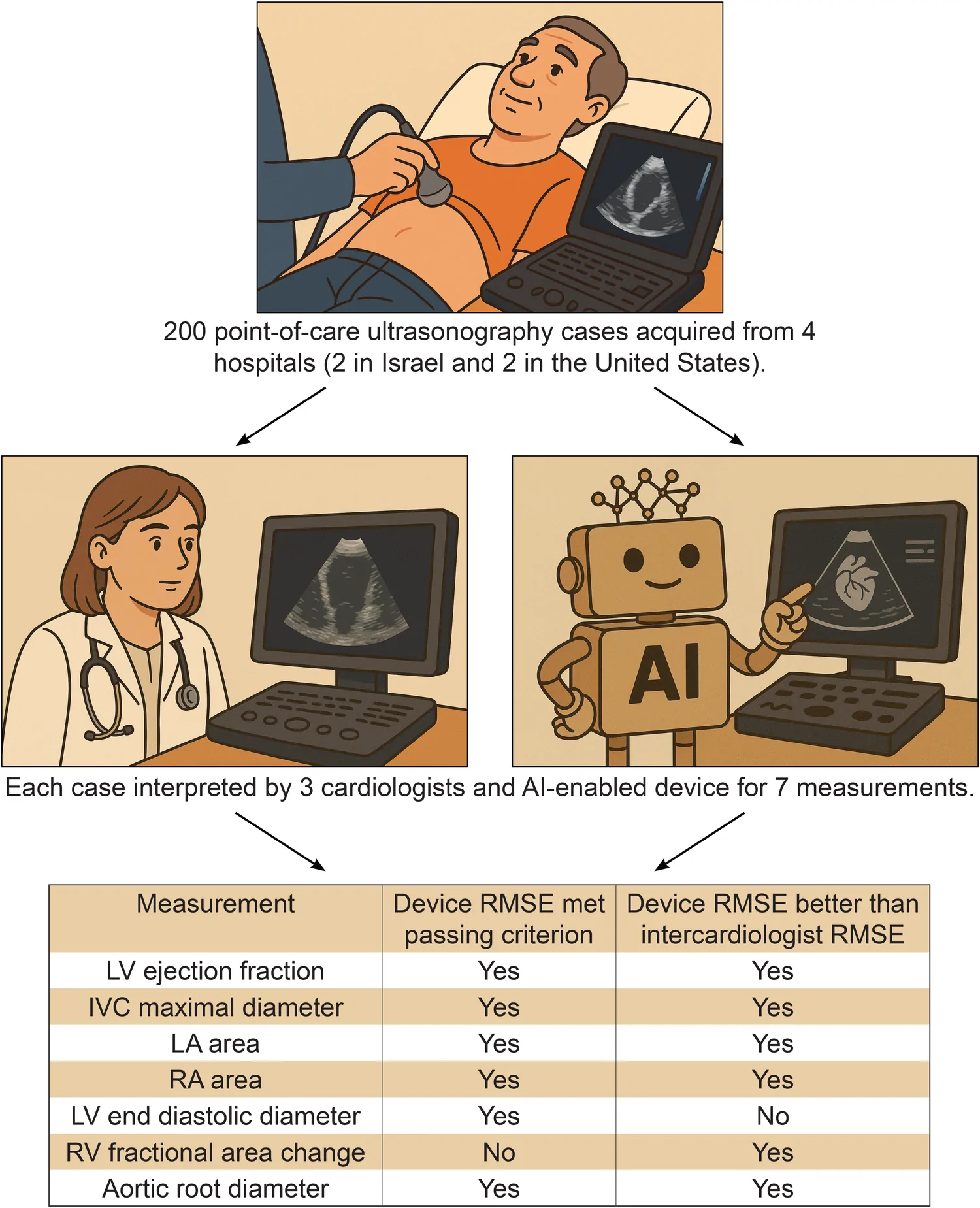
Study summary. The results summary shows the RMSE of the device compared with the average cardiologist measurement (device RMSE) and the average of the pairwise RMSEs between each combination of cardiologists (intercardiologist RMSE). The passing criteria were predefined as detailed in the Methods section. IVC, inferior vena cava; LA, left atrial; LV, left ventricular; RA, right atrial; RMSE, root mean square error; RV, right ventricular. Images generated with ChatGPT.

Overall, this performance suggests that the device provides accurate and reliable measurements. The improved RMSEs compared with cardiologists may reflect the ability of the device to average across multiple beats and loops, with the cardiologists having been asked to quantify most measurements only once. This situation, however, mirrors the real world in that cardiologists and other clinicians are often constrained by the need to manually obtain measurements, while automated measurements can leverage a greater ability to average. A similar improvement in interobserver variability has previously been shown for conventional echocardiograms (21). The ability to average may also reflect the ability of the device to learn from the measurement styles of multiple cardiologists; in the context of electroencephalogram interpretation, this use of AI to average has been described as simulating a committee of independent experts (33).

The one measurement where the device did not achieve the passing criteria was for RV fractional area change. For this measurement, however, the RMSE between the device and the cardiologists was still better than that between the cardiologists, reflecting the large variability that can be seen with this measurement and reinforcing the benefit that the device can provide through averaging. LV end-diastolic diameter was the one measurement where the RMSE was greater between the device and the cardiologists compared with that between the cardiologists, although the RMSE between the device and the cardiologists still achieved the secondary endpoint passing criterion.

POCUS provides clinicians with the ability to obtain critical diagnostic information at the bedside. For example, the knowledge of a patient's LV ejection fraction or IVC maximal diameter can guide next management steps in a critical ill patient including whether to administer fluids (34, 35). The Lumify (Philips) ultrasound probe, which was one of four assessed here, can connect directly to smart phones and tablets (25), while the Kosmos (EchoNous) ultrasound probe can connect directly to tablets (26). The use of the device with these ultrasound probes may be particularly beneficial in resource-limited settings.

A key challenge with POCUS acquisition is that the image acquisition process is often not as standardized as a conventional echocardiogram performed in an echocardiography laboratory. The reasons include that the POCUS images are often obtained in a more condensed time frame and by a provider with less training in image acquisition than an echocardiography technologist. This study assessed how the device handles this challenge in multiple ways. First, the device undergoes quality control checks to ensure sufficient image quality and the presence of key points within images; the device did not provide a quantification for between 0.5% (for LV ejection fraction) and 13.5% (for LV end-diastolic diameter) of cases for each measurement. Second, an image quality score was provided for each case; the RMSEs for image quality scores of ≤4 and >4 were similar for all measurements. Third, the RMSEs were also similar for all measurements when the person acquiring the scan was a physician or a sonographer. The first of these items also provides the benefit of avoiding erroneous outputs from the device because of insufficient image quality. In the clinical environment, this situation could prompt reacquisition of the image or even referral for a conventional echocardiogram to ensure that more diagnostically accurate measurements are obtained.

A key limitation of this study is that it was performed outside the clinical environment following the image acquisition process and did not assess the impact of the device use on patient care and outcomes. Furthermore, the study did not assess the ability to generate the measurements in real time with POCUS acquisition, where the device may provide the greatest clinical utility. Nevertheless, the images in this study were prospectively acquired at the bedside by mostly handheld devices and not as part of formal echocardiography studies obtained in an echocardiography laboratory; therefore, they represented the real-world scenario where this device would be used. Likewise, this study compared the device directly with three ground truth cardiologists to establish whether it exhibited similar performance for the specified measurements; it did not aim to look at cross-modality performance such as comparing the device performance on POCUS with physiologic measurements on a conventional echocardiogram.

This study was limited to four sites and the device will inevitably encounter new scenarios with clinical use, including more ultrasound probe manufacturers and models. The study was also not powered for individual subgroup analyses and no adjustment was made for multiple comparisons; therefore, the subgroup findings should be interpreted as exploratory in nature, with the confidence intervals reflecting the uncertainty around the point estimates. For these reasons, the ongoing assessment of the device as it is deployed in the clinical environment, especially at additional sites that did not contribute to training data, will be critical in strengthening the confidence of its performance. Furthermore, future studies will be needed to assess the impact of the device on clinical decisions and outcomes as it provides the measurements in real time with POCUS acquisition.

## Conclusion

The AI-enabled device in this study accurately calculated seven cardiac measurements on POCUS, especially when benchmarked against intercardiologist variability. It could facilitate better clinical decision-making, including in the acute care environment. Further studies will be required to assess its performance in the real-world setting, including its impact on clinical decision-making.

## Data Availability

The data that support the findings of this study are not publicly available. The data may be available from the corresponding author upon reasonable request. Requests to access the datasets should be directed to James Hillis, james.hillis@mgh.harvard.edu.
