# Supplementary material for: Automated artificial intelligence–enabled measurement of cardiac structures on point-of-care ultrasonography: a prospective multicenter validation study
Source: Front Cardiovasc Med. 2026 Sep 21;13:1891962. doi: 10.3389/fcvm.2026.1891962 (PMC13635147; doi:10.3389/fcvm.2026.1891962)
Supplement: Supplementary file 2 [file Table1.docx]

**Supplementary Table 1: Views used to obtain each measurement.** Abbreviations: A4, apical four chamber; PLAX, parasternal long axis.

| **Measurement** | **View** |
| --- | --- |
| Left ventricular ejection fraction | A4 / PLAX |
| Inferior vena cava maximal diameter | Subcostal |
| Left atrial area | A4 |
| Right atrial area | A4 |
| Left ventricular end diastolic diameter | PLAX |
| Right ventricular area (end of diastole and end of systole); to calculate right ventricular fractional area change | A4 |
| Aortic root diameter | PLAX |

**Supplementary Table 2: Excluded cases.** The cases were excluded when the cardiologists could not obtain the measurement or the device outputted “NULL” (see Methods for explanation). If a row exceeds a total of 200 cases, it indicates that a case was both excluded by the cardiologists and received a “NULL” device output. Abbreviations: IVC, inferior vena cava; LA, left atrial; LV, left ventricular; RA, right atrial; RV, right ventricular.

|  | Cardiologists could not obtain | “NULL” device output | Final cohort |
| --- | --- | --- | --- |
| LV ejection fraction | 2 (1.0%) | 1 (0.5%) | 197 |
| IVC maximal diameter | 16 (8.0%) | 25 (12.5%) | 175 |
| LA area | 3 (1.5%) | 10 (5.0%) | 188 |
| RA area | 5 (2.5%) | 18 (9.0%) | 178 |
| LV end diastolic diameter | 1 (0.5%) | 27 (13.5%) | 173 |
| RV fractional area change | 25 (12.5%) | 18 (9.0%) | 161 |
| Aortic root diameter | 1 (0.5%) | 2 (1.0%) | 197 |

**Supplementary Table 3: Subgroup error analysis.** The number and percentage of cases with values within or outside the 95% LOA based on the Bland-Altman analysis for different subgroups. The subgroups were for LV ejection fraction, ultrasound probe manufacturer and image quality score. The Bland-Altman analysis was performed as described in the Methods. *Cardiologist measurements for LV ejection fraction were not available for 2 cases (see Supplementary Table 2), resulting in possible differences in the total N for those rows. Abbreviations: IVC, inferior vena cava; LA, left atrial; LOA, limits of agreement; LV, left ventricular; N, number of cases; RA, right atrial; RV, right ventricular.

|  | LV ejection fraction | | IVC maximal diameter | | LA area | | RA area | | LV end diastolic diameter | | RV fractional area change | | Aortic root diameter | |
| --- | --- | --- | --- | --- | --- | --- | --- | --- | --- | --- | --- | --- | --- | --- |
|  | Within 95% LOA  (N=185) | Outside 95% LOA  (N=12) | Within 95% LOA  (N=167) | Outside 95% LOA  (N=8) | Within 95% LOA  (N=178) | Outside 95% LOA  (N=10) | Within 95% LOA  (N=168) | Outside 95% LOA  (N=10) | Within 95% LOA  (N=161) | Outside 95% LOA  (N=12) | Within 95% LOA  (N=153) | Outside 95% LOA  (N=8) | Within 95% LOA  (N=186) | Outside 95% LOA  (N=11) |
| LV ejection fraction* |  |  |  |  |  |  |  |  |  |  |  |  |  |  |
| <30% | 18  (9.7%) | 1  (8.3%) | 15  (9.0%) | 1  (12.5%) | 15  (8.4%) | 3  (30.0%) | 17  (10.1%) | 0  (0.0%) | 14  (8.7%) | 0  (0.0%) | 13  (8.5%) | 1  (12.5%) | 19  (10.2%) | 0  (0.0%) |
| ≥30% to <53% | 41  (22.2%) | 8  (66.7%) | 41  (24.6%) | 0  (0.0%) | 46  (25.8%) | 0  (0.0%) | 43  (25.6%) | 2  (20.0%) | 43  (26.7%) | 3  (25.0%) | 36  (23.5%) | 2  (25.0%) | 44  (23.7%) | 5  (45.5%) |
| ≥53% | 126  (68.1%) | 3  (25.0%) | 110  (65.9%) | 7  (87.5%) | 116  (65.2%) | 7  (70.0%) | 107  (63.7%) | 8  (80.0%) | 102  (63.4%) | 9  (75.0%) | 104  (68.0%) | 5  (62.5%) | 121  (65.1%) | 6  (54.5%) |
|  |  |  |  |  |  |  |  |  |  |  |  |  |  |  |
| Ultrasound probe manufacturer (model) |  |  |  |  |  |  |  |  |  |  |  |  |  |  |
| EchoNous (Kosmos) | 35  (18.9%) | 2  (16.7%) | 32  (19.2%) | 2  (25.0%) | 34  (19.1%) | 2  (20.0%) | 34  (20.2%) | 0  (0.0%) | 28  (17.4%) | 4  (33.3%) | 25  (16.3%) | 0  (0.0%) | 35  (18.8%) | 2  (18.2%) |
| Philips (Lumify) | 73  (39.5%) | 7  (58.3%) | 68  (40.7%) | 2  (25.0%) | 72  (40.4%) | 2  (20.0%) | 67  (39.9%) | 4  (40.0%) | 64  (39.8%) | 6  (50.0%) | 64  (41.8%) | 2  (25.0%) | 74  (39.8%) | 6  (54.5%) |
| Wisonic (Navi) | 22  (11.9%) | 3  (25.0%) | 23  (13.8%) | 0  (0.0%) | 24  (13.5%) | 1  (10.0%) | 22  (13.1%) | 2  (20.0%) | 17  (10.6%) | 1  (8.3%) | 21  (13.7%) | 1  (12.5%) | 24  (12.9%) | 1  (9.1%) |
| GE Healthcare (Venue) | 55  (29.7%) | 0  (0.0%) | 44  (26.3%) | 4  (50.0%) | 48  (27.0%) | 5  (50.0%) | 45  (26.8%) | 4  (40.0%) | 52  (32.3%) | 1  (8.3%) | 43  (28.1%) | 5  (62.5%) | 53  (28.5%) | 2  (18.2%) |
|  |  |  |  |  |  |  |  |  |  |  |  |  |  |  |
| Image quality score |  |  |  |  |  |  |  |  |  |  |  |  |  |  |
| ≤4 | 46  (24.9%) | 2  (16.7%) | 39  (23.4%) | 2  (25.0%) | 42  (23.6%) | 3  (30.0%) | 38  (22.6%) | 3  (30.0%) | 41  (25.5%) | 3  (25.0%) | 37  (24.2%) | 2  (25.0%) | 45  (24.2%) | 3  (27.3%) |
| >4 | 95  (51.4%) | 4  (33.3%) | 83  (49.7%) | 6  (75.0%) | 88  (49.4%) | 6  (60.0%) | 87  (51.8%) | 5  (50.0%) | 81  (50.3%) | 5  (41.7%) | 76  (49.7%) | 6  (75.0%) | 94  (50.5%) | 4  (36.4%) |
| Unknown | 44  (23.8%) | 6  (50.0%) | 45  (26.9%) | 0  (0.0%) | 48  (27.0%) | 1  (10.0%) | 43  (25.6%) | 2  (20.0%) | 39  (24.2%) | 4  (33.3%) | 40  (26.1%) | 0  (0.0%) | 47  (25.3%) | 4  (36.4%) |
